# Assessing the Spectral Characteristics of Dye- and Pigment-Based Inkjet Prints by VNIR Hyperspectral Imaging

**DOI:** 10.3390/s22020603

**Published:** 2022-01-13

**Authors:** Lukáš Krauz, Petr Páta, Jan Kaiser

**Affiliations:** 1 Department of Radioelectronics, Faculty of Electrical Engineering, Czech Technical University in Prague, Technická 2, 166 27 Prague, Czech Republic; pata@fel.cvut.cz (P.P.); kaiser@fomei.com (J.K.); 2 FOMEI s.r.o., U Libeňského pivovaru 2015, 180 00 Prague, Czech Republic

**Keywords:** hyperspectral imaging, VNIR, inkjet printing, dyes, pigments, photo paper, archiving

## Abstract

Fine art photography, paper documents, and other parts of printing that aim to keep value are searching for credible techniques and mediums suitable for long-term archiving purposes. In general, long-lasting pigment-based inks are used for archival print creation. However, they are very often replaced or forged by dye-based inks, with lower fade resistance and, therefore, lower archiving potential. Frequently, the difference between the dye- and pigment-based prints is hard to uncover. Finding a simple tool for countrified identification is, therefore, necessary. This paper assesses the spectral characteristics of dye- and pigment-based ink prints using visible near-infrared (VNIR) hyperspectral imaging. The main aim is to show the spectral differences between these ink prints using a hyperspectral camera and subsequent hyperspectral image processing. Two diverse printers were exploited for comparison, a hobby dye-based EPSON L1800 and a professional pigment-based EPSON SC-P9500. The identical prints created via these printers on three different types of photo paper were recaptured by the hyperspectral camera. The acquired pixel values were studied in terms of spectral characteristics and principal component analysis (PCA). In addition, the obtained spectral differences were quantified by the selected spectral metrics. The possible usage for print forgery detection via VNIR hyperspectral imaging is discussed in the results.

## 1. Introduction

Currently, there is a vast range of scientific and industrial fields where hyperspectral imaging (HSI) finds its usage. Among those fields, plant [1] and soil [2] monitoring, agriculture [3], medicine [4,5], food analysis [6], remote sensing [7], forensics [8,9,10], or cultural heritage [11] can be counted. In general, HSI can be applied in a variety of spectral bands. There are HSI applications in the short-wave infrared (SWIR) 1–3 μm [12], mid-wave infrared (MWIR) 3–5 μm [13], and long-wave infrared (LWIR) 8–14 μm [14] bands. Nevertheless, the applications of HSI in visible near-infrared (VNIR) 0.3–1 μm spectral band are most frequent [15,16]. Concerning specifically cultural heritage, HSI has found its utilization in historical painting analysis [17,18,19], art forgery detection [20], and painting restoration [21].

Recently, there has also been a high demand for fine art photography, but also for document printing, which requires a perfect color representation and archiving parameters. This segment of art and document printing is filled by a variety of photo printers fitting the mentioned requirements. Two dominant types of printing techniques are dye and inkjet printing [22]. In general, for art printing and archiving, inkjet printing plays a major role. There are inkjet printers based on two dominant ink approaches, dye-based and pigment-based. However, each type is suitable for different applications. Less expensive dye-based printers find their place among the common customers for hobby printing purposes. On the contrary, pigment-based printers provide a professional printing solution included in specialized printing labs, and they are suitable particularly for documents, photographs, and fine art printing purposes with the emphasis on archiving [23,24]. Frequently, due to a reduction in costs, pigment-based prints are replaced or forged by dye-based prints that are less suitable for archiving. Despite the significant improvements in the dye-based printing technology, current dye-based prints still reach only a quarter lightfastness and fade resistance compared to pigment-based prints. Moreover, due to the cutting-edge dye-based printing technology and its broader color gamut, a dye-based forged print can be easily mistaken for pigment-based prints and remain unrecognized.

Although both types of ink are used for highly dissimilar purposes, they may be easily interchanged and unidentified by the naked eye, even by professionals. Nevertheless, it is well known that dye- and pigment-based inks are water-based inks, but consist of different technologies of color creation. Therefore, the spectral characteristics may also behave differently, even if the dye- and pigment-based prints appear similar. To assess the spectral characteristics of the dye- and pigment-based inks across the whole print and to identify the origin printer, HSI may become a useful and straightforward tool. There have also been some approaches focused on the color measurement [25] and spectral analysis of dyes [26], pigments [27,28], and ink-printed documents [29]. However, direct spectral comparisons that aim at dye- and pigment-based inkjet print differences have not been performed. There are also other techniques for art analysis and forgery identification such as simple microscopy, mass spectrometry, colorimetry, standard reflectance spectroscopy, chemical analysis, and X-ray methods [30]. However, some of these techniques may not be suitable for inkjet print evaluation. Compared to the mentioned approaches, HSI provides a fast, simple, and non-invasive method for thorough spectral analysis across the whole print, which favors its usage.

This paper focuses on the VNIR HSI assessment of dye- and pigment-based prints, their spectral comparison, and the quantification of their spectral differences. To eliminate the spectral effects of the photo paper, the prints were assessed on three different types of paper. For the assessment, various spectral similarity metrics were used, as well as principal component analysis (PCA) of the hyperspectral (HS) image data. Therefore, the main goal was the verification that VNIR HSI may be used for validation and identification of whether the print is made by a professional and long-lasting pigment-based printer with archive potential or by a hobby dye-based printer.

The paper is structured into five main sections. The Introduction is followed by the section on the color dye- and pigment-based printing and hyperspectral reflectance representation. The following section describes the exploited methods, algorithms, and the HS data acquisition process. The fourth section is focused on the obtained results’ presentation and subsequent discussion. The last section provides the overall conclusion.

## 2. Print Color Management and Hyperspectral Representation

As was mentioned in the Introduction, among the inkjet printers, dye- and pigment-based inks are the two main types of inks used for photograph printing purposes. In general, both ink types fall under the set of water-based or aqueous inks. Dye-based inks are homogeneous color fluids with fully dissoluble dyes. They are usually used in hobby inkjet printers due to their lower manufacturing costs. Dye-based inks also do not provide such lightfastness and fade resistance as pigment-based inks. On the contrary, dye-based inks usually offer a broader color gamut than pigment-based ones, especially on glossy papers. Usually, the dye-based inkjet printers contain six separate inks. Except for the standard cyan, magenta, yellow, black (CMYK) configuration, these printers have two additional colors, light cyan and light magenta.

On the contrary, pigment-based inks consist of microscopic color grains inserted in the sheer liquid. These inks are exploited in professional inkjet printers and labs due to their superb lightfastness. Therefore, pigment-based inks are often used for fine art printing purposes, photo archiving, and large format photography. Typically, the pigment-based printers include nine different inks, but very often, there are several more inks included. High-level pigment-based printers standardly contain a 12-ink set. Two types of black inks (selected according to the print purpose) are appended by cyan, light cyan, yellow, vivid magenta, vivid light magenta, violet, orange, green, grey, and light grey.

Not only the different characteristics of the presented inks and printers, but also the type of photo paper may play a significant role in the printed photograph’s lightfastness and appearance. There are several types of photo paper suitable for various applications with matte, semiglossy, or glossy surfaces. They may also be created from a variety of materials, such as standard resin-coated (RC) paper, cotton paper, baryte paper, and others.

All the influences mentioned above affect the final parameters of photograph prints. Although the dye- and pigment-based colors in the printed photograph may appear similar, their spectral characteristics may significantly vary. Measuring the simple spectral signature of a single spatial point of the print, standard spectroscopy techniques can be used. However, aiming for a more complex ink spectral analysis across the whole printed image, the hyperspectral imaging tool can be exploited. Assume a printed photograph, either dye- or pigment-based, subjected to a source of radiation. Such radiation then may be absorbed, transmitted, or reflected from the photograph. For the spectral measurement, the amount of reflected light captured by the HS camera is the most critical parameter. Let an illumination with defined spectral characteristics $\phi_{i}\left(\lambda \right)$ be described as incident flux on the object, where λ represents the spectral wavelength. Similarly, assume a flux reflected from the object defined as $\phi_{r}\left(\lambda \right)$. The reflectance then may be expressed as:

(1) $$R\left(\lambda \right)=\frac{\phi_{r}\left(\lambda \right)}{\phi_{i}\left(\lambda \right)}.$$

**Figure 1:**
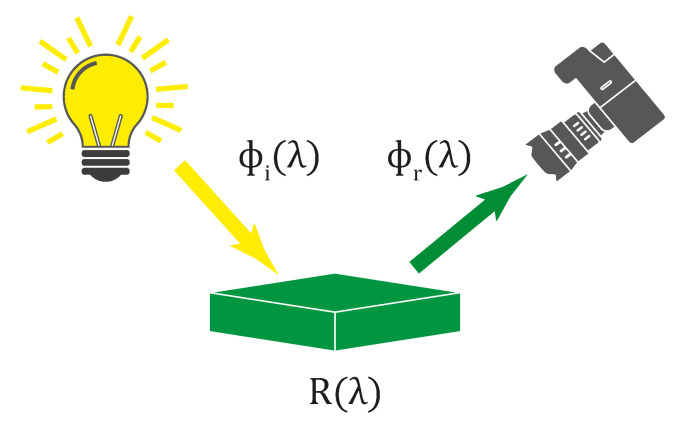
Illustration of spectral reflectance depending on the illumination.

This process is represented by Figure 1. For simplicity, the specular reflection with particular angle θ is assumed. In the case of the the HS measurement of the photo-printed inks, the overall photo reflectance $R\left(\lambda \right)$ consists of the characteristics of the underlay paper $R_{paper}\left(\lambda \right)$, as well as single or mixed printer color inks $R_{ink}\left(\lambda ,c\right)$, where *c* represents the color mixture index. In a simplified way, the reflected light flux $\phi_{r}\left(\lambda \right)$ consists of two parts. The first one:

(2) $$\phi_{r}^{\prime }\left(\lambda \right)=\phi_{i}\left(\lambda \right)\cdot R_{ink}\left(\lambda ,c\right)$$

is the directly reflected light flux from the ink layer. The second one:

(3) $$\phi_{r}^{\prime \prime }\left(\lambda \right)=\phi_{i}\left(\lambda \right)\cdot R_{paper}\left(\lambda \right)\cdot T_{ink}^{2}\left(\lambda ,c\right)$$

is the part of the flux reflected from the surface of the photo paper after propagation through the ink layer, where $T_{ink}^{2}\left(\lambda ,c\right)$ is the transmission of the ink mixture layer. The overall reflected flux is:

(4) $$\phi_{r}\left(\lambda \right)=\phi_{i}\left(\lambda \right)\cdot \left[R_{paper}\left(\lambda \right)\cdot T_{ink}^{2}\left(\lambda ,c\right)+R_{ink}\left(\lambda ,c\right)\right].$$

Therefore, the reflectance of the printed image should be represented as:

(5) $$R\left(\lambda \right)=R_{paper}\left(\lambda \right)\cdot T_{ink}^{2}\left(\lambda ,c\right)+R_{ink}\left(\lambda ,c\right).$$

With the exploitation of the HS camera, the additional two spatial $x_{s},y_{s}$ dimensions may be also added to the above-described spectral measurement. The reflected light from the object $\phi_{r}\left(\lambda \right)$ with the spatial coordinates $x_{s},y_{s}$ may be acquired and transformed into the HS 3D image (hypercube) $I_{HS}\left(x_{s},y_{s},\lambda \right)$ considering the HS camera sensitivity $\psi \left(x_{s},y_{s},\lambda \right)$ as:

(6) $$\phi_{r}\left(x_{s},y_{s},\lambda \right)⟶I_{HS}\left(x_{s},y_{s},\lambda \right),$$

where:

(7) $$I_{HS}\left(x_{s},y_{s},\lambda \right)=\phi_{i}\left(x_{s},y_{s},\lambda \right)R\left(x_{s},y_{s},\lambda \right)\psi \left(x_{s},y_{s},\lambda \right).$$

Thus, the obtained hypercube can be processed and analyzed in terms of the particular spectral reflectance for each obtained pixel of the captured photograph print.

## 3. Methods and Algorithms

The key decision for the dye- and pigment-based ink print comparison was the selection of the inkjet printer representatives. In the first place, the hobby inkjet printer EPSON L1800 with 6 color inks was selected as a dye-based representative. Complementarily, the professional inkjet printer EPSON SC-P9500 equipped with 12 diverse pigment inks was chosen. To cover the different possibilities of ink and paper chemical interactions, and therefore variations in measured reflectance, 3 types of distinct photo papers with size A4 were selected. Each photo paper was selected on purpose as the most common representative from a different application photo paper class. The most commonly exploited photo papers from diverse application classes were selected as representatives. The first photo paper was semigloss FOMEI Archival Velvet with a weight of 265 g/m${}^{2}$, which contains a small amount of optical brighteners and is designed for hobby and professional archiving purposes. The second selected photo paper was also semigloss FOMEI PRO Pearl with the same weight of 265 g/m${}^{2}$. This paper contains a significant amount of optical brighteners and is most frequently used among amateur, but also professional photographers. The last selected paper was a matte-textured paper FOMEI Cotton Textured that weighs 240 g/m${}^{2}$ and is made from 100% cotton. This art inkjet photo paper does not contain any optical brighteners, is acid free, and is primarily used for fine art applications.

**Figure 2:**
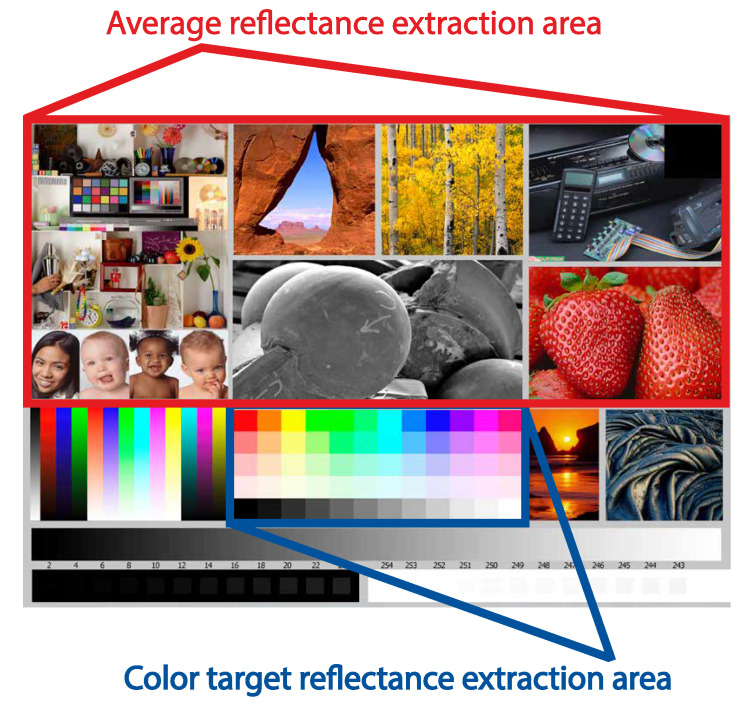
The selected test image (created by Bill Atkinson, Jack Flesher, and Uwe Steinmueller) for dye- and pigment-based prints [31]. This image contains a series of varying scenes, as well as printed color targets that may be easily used for the reflectance analysis. The red rectangle highlights the image area that was used for the overall average reflectance analysis. The blue rectangle highlights the image area used for the reflectance extraction of specific color samples.

Furthermore, as an example image, the standardized test image from [31] was selected. This test image allows a comprehensive evaluation of diverse colors and scenes with changing patterns. Therefore, the prints of this image are suitable for ink reflectance analysis. The test image with highlighted areas used for a detailed reflectance analysis can be seen in Figure 2.

Thus, all dye- and pigment-based images printed on all the above-mentioned photo papers were recaptured by the hyperspectral camera and further analyzed. The acquisition and overall hyperspectral image data processing are described in the following sections.

### 3.1. Acquisition

**Figure 3:**
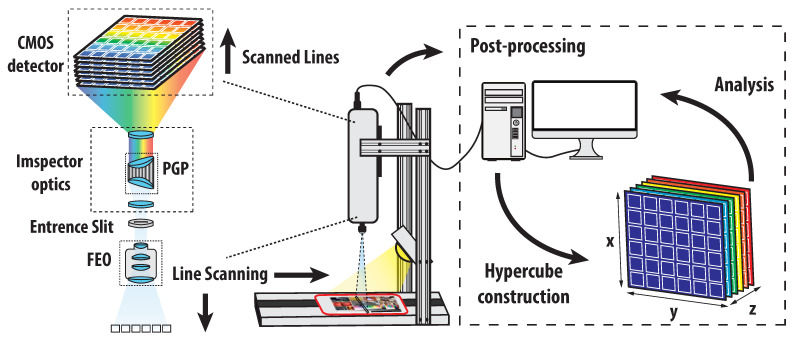
Hyperspectral image capturing and hypercube construction.

The dye- and pigment-based prints were scanned by the hyperspectral camera SPECIM PFD4K-65-V10E. This camera type is based on the pushbroom line scanning principle. The scanned object was placed on the movable table under the fore camera optics. The table was then illuminated by the halogen lamp connected to the whole table setup. After adjusting the suitable movement speed of the table, the moving object was scanned line by line until the full scan was finished. During the scanning process, each scanned line or, more precisely, each pixel was decomposed into the whole spectral range of the camera. For this purpose, some dispersion components such as prisms, diffraction grating, or their combination were used among the camera internal parts. The full hyperspectral scan was then digitally processed, and the hypercube with all merged hyperspectral lines was constructed. The whole scanning principle is outlined in Figure 3.

The overall spectral range of the SPECIM hyperspectral camera covers the VNIR spectral band 400–1000 nm. The camera provides decomposition into 768 separate spectral channels. The FWHM spectral resolution corresponds to 3 nm. The camera is equipped with a fore optics OLE 23, with focal length 23 mm, f-number f/2.4, and transparency in the whole VNIR spectral band. The remaining parameters of the camera are outlined in Table 1. All captured HS images of the prints were taken with the following settings: a frame rate equal to 25 Hz and exposition equal to 39 ms.

### 3.2. Image Data Pre-Processing

Let a hyperspectral image captured directly from the above-described HS system be represented as a hypercube. To minimize the noise influence of the camera components and illumination effects, radiometric calibration is necessary. According to Equation (7), assume the hypercube consisting of matrix slices $I_{HS}\left(x,y,z\right)$, where indices x=1,⋯,H and y=1,⋯,W represent the spatial dimension of the image (see Figure 3). The last symbol z=1,⋯,S is the wavelength index of each matrix slice depending on the operational spectral range of the HS system. During the hypercube capturing process, several dark and white reference images were recorded. The dark image was usually taken with a closed camera shutter as a sequence of images. The dark frame is represented by the pushbroom system as $I_{DARK}\left(i,y,z\right)$, where i=1,…,N, and N is the number of dark frames taken. The white reference spectrum image was taken by capturing an image sequence of the calibration target with known reflectance (typically 99 %). Usually, for pushbroom systems, several spectral images of one spatial line are acquired. The white reference image is then represented as $I_{WHITE}\left(i,y,z\right)$, where i=1,…,M and *M* also corresponds to the number of frames taken, similar as for the dark image. Due to the matrix mismatch between the captured hypercube, dark image, and white image, the resulting calibrated hypercube with normalized reflectance must be evaluated for each row of the hypercube $I_{r}\left(y,z\right)$ separately as:

(8) $$\begin{array}{c} I_{D}\left(y,z\right)=\frac{1}{N}\sum_{i=1}^{N}I_{DARK}\left(i,y,z\right), \end{array}$$

(9) $$\begin{array}{c} I_{W}\left(y,z\right)=\frac{1}{M}\sum_{i=1}^{M}I_{WHITE}\left(i,y,z\right), \end{array}$$

(10) $$\begin{array}{c} I_{r}\left(y,z\right)=\frac{I_{HS}\left(y,z\right)-I_{D}\left(y,z\right)}{I_{W}\left(y,z\right)-I_{D}\left(y,z\right)}. \end{array}$$

The whole normalized hypercube $I_{norm}\left(x,y,z\right)$ was obtained by stacking the calibrated rows $I_{r}\left(y,z\right)$ in the original hypercube shape. Even though radiometric calibration was performed, the calibrated hyperspectral image usually still contained spectral noise. Therefore, a standard Savitzky–Golay [32] filtering was performed. For this purpose, the third-order polynomial with a 15-point window was selected [33]. The Savitzky–Golay method was separately applied to each hyperspectral pixel, filtering only the spectral dimension. The filtering process [34] can be expressed as:

(11) $$I^{*}\left(z\right)=\frac{\sum_{k=-m}^{k=m}c_{k}I\left(z+k\right)}{2m+1},$$

where $I^{*}\left(z\right)$ corresponds to one spectral data point of the selected pixel, *m* is half of the filter window, and $c_{k}$ is the filter convolution coefficient for the k-th filtering. Then, the key step of the filtering is fitting the original spectral data points by the n-th order polynomial in a defined sliding window via the least-squares estimation.

### 3.3. Spectral Similarity Measures

With a spectral response (reflectance) for all pixels from the captured dye- and pigment-based prints, it is possible to quantify the spectral differences by various spectral similarity measures. Assume a pre-processed HS image of either a dye- or pigment-based test image print. Furthermore, suppose that the average reflectance $\bar{R}\left(z\right)$ of the selected HS image pixel area $I_{sub}\left(x^{\prime },y^{\prime },z\right)$ is expressed as:

(12) $$\bar{R}\left(z\right)=\frac{1}{H^{\prime }W^{\prime }}\sum_{x^{\prime }=1}^{H^{\prime }}\sum_{y^{\prime }=1}^{W^{\prime }}I_{sub}\left(x^{\prime },y^{\prime },z\right),$$

where indices $x^{\prime }=1,\cdots ,H^{\prime }$ and $y^{\prime }=1,\cdots ,W^{\prime }$ correspond to the height and width of the selected subimage pixel area.

One of the fundamental metrics for the comparison of two reflectance $\bar{R_{1}},\bar{R_{2}}$ is the spectral angle mapper (SAM) [35]. It can be written as:

(13) $${\mathrm{SAM}}_{\bar{R_{1}},\bar{R_{2}}}=\cos^{-1}\left(\frac{\sum_{z=1}^{S}\bar{R_{1}}\left(z\right)\bar{R_{2}}\left(z\right)}{\sqrt{\sum_{z=1}^{S}\bar{R_{1}}{\left(z\right)}^{2}}\sqrt{\sum_{z=1}^{S}\bar{R_{2}}{\left(z\right)}^{2}}}\right).$$

The other metric that may be used for reflectance comparison is the spectral information divergence (SID) [36]. This metric returns the divergence between two probability distributions extracted from two spectral signatures defined as:

(14) $$D_{1}\left(z\right)=\bar{R_{1}}\left(z\right)/\sum_{z=1}^{S}\bar{R_{1}}\left(z\right),$$

(15) $$D_{2}\left(z\right)=\bar{R_{2}}\left(z\right)/\sum_{z=1}^{S}\bar{R_{2}}\left(z\right).$$

The SID is then represented as:

(16) $${\mathrm{SID}}_{\bar{R_{1}},\bar{R_{2}}}=\sum_{z=1}^{S}D_{2}\left(z\right)\log\left(\frac{D_{2}\left(z\right)}{D_{1}\left(z\right)}\right)+\sum_{z=1}^{S}D_{1}\left(z\right)\log\left(\frac{D_{1}\left(z\right)}{D_{2}\left(z\right)}\right).$$

Having defined the fundamental spectral similarity metrics, the combined hybrid methods may be introduced. From the definition above, the hybrid method the spectral information divergence spectral angle mapper (SIDSAM) [37] can be expressed as:

(17) $${\mathrm{SIDSAM}}_{\bar{R_{1}},\bar{R_{2}}}={\mathrm{SID}}_{\bar{R_{1}},\bar{R_{2}}}\times \tan\left({\mathrm{SAM}}_{\bar{R_{1}},\bar{R_{2}}}\right).$$

Another and more advanced hybrid spectral similarity metric for reflectance comparison is Jeffries–Matusita spectral angle mapper (JMSAM) [38]. Similar to the SIDSAM, the JMSAM involves the fusion of two metrics, the JM distance and the SAM. The JM distance:

(18) $${\mathrm{JM}}_{\mathrm{dist}}=2\left(1-\exp\left(-BD_{1,2}\right)\right)$$

involves a computation of the Bhattacharyya distance $BD_{\bar{R_{1}},\bar{R_{2}}}$ of two compared spectral reflectances as:

(19) $$\begin{array}{c} BD_{\bar{R_{1}},\bar{R_{2}}}=\frac{1}{8}{\left(\mu_{1}-\mu_{2}\right)}^{⊺}{\left(\frac{\sigma_{1}+\sigma_{2}}{2}\right)}^{-1}\left(\mu_{1}-\mu_{2}\right)\frac{1}{2}\ln\left(\frac{\det\left(\frac{\sigma_{1}+\sigma_{2}}{2}\right)}{\sqrt{\det\sigma_{1}\det\sigma_{2}}}\right), \end{array}$$

where $\mu_{1},\mu_{2}$ are the mean values of the compared reflected spectra $\sigma_{1},\sigma_{2}$ and their variance. The JMSAM then can be computed as:

(20) $${\mathrm{JMSAM}}_{\bar{R_{1}},\bar{R_{2}}}={\mathrm{JM}}_{\mathrm{dist}}\times \tan\left({\mathrm{SAM}}_{\bar{R_{1}},\bar{R_{2}}}\right).$$

Each of the presented measures gives a particular score assessing the similarity between the reflectance spectra. In general, a lower score of these metrics indicates a strong match between the compared spectral signatures. For the purpose of this work, the hybrid JMSAM and SIDSAM metrics were selected as representatives of the comparison of the dye- and pigment-based ink prints.

### 3.4. Principle Component Analysis

In general, the captured HS image often includes redundancy in the neighbor spectral bands and, therefore, a high correlation between them. Techniques such as PCA allow decorrelating the whole HS image into a set of new orthogonal bases, also known as principal components (PCs). The first few principal components usually contain the most captured variance of the original HS image, and therefore, this HS image can be described by only a few PCs.

Assume the original HS image $I\left(x,y,z\right)$ reshaped into a 2D matrix $X\left(i,z\right)$, where indices i=1,⋯,HW and z=1,⋯,S. This matrix is then mean-centered as:

(21) $$X^{\prime }\left(i,z\right)=X\left(i,z\right)-\frac{1}{HW}\sum_{i=1}^{HW}X\left(i,z\right).$$

Subsequently, the matrix $X^{\prime }\left(i,z\right)$ may be decomposed by the singular-value decomposition (SVD) method [39] as:

(22) $$X^{\prime }\left(i,z\right)=U\left(i,i\right)S\left(i,z\right)V^{⊺}\left(z,z\right),$$

where $U\left(i,i\right)$ and $V^{⊺}\left(z,z\right)$ are unitary matrices carrying the principal components and eigenvectors, respectively. Diagonal matrix $S\left(i,z\right)$ holds the singular values. For the following operations, the reduction of the matrix $U\left(i,i\right)$ to $U\left(i,z\right)$ is eligible. After the extraction of diagonal values from $S\left(i,z\right)$ into a vector $s\left(z\right)$, the eigenvalues can be obtained from the expression:

(23) $$\Lambda \left(z\right)=\frac{s^{2}\left(z\right)}{HW-1},$$

where Λ$\left(z\right)$ is a vector of all eigenvalues. The transformation of the original matrix $X^{\prime }\left(i,z\right)$ into the new orthogonal coordinate system based on the $I\left(x,y,z\right)$ values may be calculated according to the following expression:

(24) $$X_{t}\left(i,z\right)=U\left(i,z\right)s^{⊺}\left(z\right).$$

Nevertheless, the index *z* does not now correspond to the wavelength index, but to the PC index in the new coordinate system.

The explained variance for each generated PC can be computed by the expression:

(25) $$\sigma^{2}\left(z\right)=\frac{100\cdot \Lambda \left(z\right)}{\sum_{z=1}^{S}\Lambda \left(z\right)},$$

where every element of vector $\sigma^{2}\left(z\right)$ corresponds to the explained variance of the particular PC. Due to the nature of the PCA, the transformed image may be usually expressed with a reduced dimension of only some PCs that cover more than 95% or 99% of the cumulative variance of the original image. The index z=1,⋯,S may be from the maximal number of PCs *S* reduced to the specified number $S^{\prime }$.

It is apparent that the the PCA method is data dependent. However, it is possible to express a different hyperspectral image using a set of PC components, or more precisely eigenvectors, derived from the PCA method applied to another HS image. Let a new HS image $I_{new}\left(x^{\prime \prime },y^{\prime \prime },z\right)$ be reshaped into matrix $N(i^{\prime \prime },z)$ for indices $i^{\prime \prime }=1,\cdots ,H^{\prime \prime }W^{\prime \prime }$ and z=1,⋯,S depending on the spatial dimension of the new HS image. Then, suppose the previously generated diagonal eigenvector matrix $V\left(z,z\right)$ of the original HS image $I\left(x,y,z\right)$ from Expression (22). The matrix $N\left(i^{\prime \prime },z\right)$ then can be transferred to the coordinate system dependent on the original HS image $I\left(x,y,z\right)$ data values as:

(26) $$N^{\prime }\left(i^{\prime \prime },z\right)=N\left(i^{\prime \prime },z\right)-\frac{1}{HW}\sum_{i=1}^{HW}X\left(i,z\right),$$

(27) $$N_{t}\left(i^{\prime \prime },z\right)=N^{\prime }\left(i^{\prime \prime },z\right)V\left(z,z\right).$$

Thus, the pixel values of the original $X_{t}\left(i,z\right)$ and new test HS image $N_{t}\left(i^{\prime \prime },z\right)$ may be compared in one coordinate system created from the original HS image. To quantify the differences between the pixel values in this coordinate system, the standard Euclidean distance can be exploited. Suppose a subset of pixel values extracted from $X_{t}\left(i,z\right)$ labeled as $P\left(i_{s},z\right)$, where $i_{s}=1,\cdots ,K$ is the pixel subset index. Similarly, assume a subset of pixels of the transformed image $N_{t}\left(i^{\prime \prime },z\right)$ represented as $Q\left(i_{s},z\right)$ with pixel indexation $i_{s}=1,\cdots ,L$. Then, the mean value vector across the selected PC dimensions for both defined subsets is given as:

(28) $$\begin{array}{c} \bar{p}\left(z\right)=\frac{1}{K}\sum_{i_{s}=1}^{K}P\left(i_{s},z\right), \end{array}$$

(29) $$\begin{array}{c} \bar{q}\left(z\right)=\frac{1}{L}\sum_{i_{s}=1}^{L}Q\left(i_{s},z\right). \end{array}$$

Analogically, the standard deviation vectors of these pixel subsets are:

(30) $$\begin{array}{c} \sigma_{p}\left(z\right)=\sqrt{\frac{1}{K}\sum_{i_{s}=1}^{K}{\left(P\left(i_{s},z\right)-\bar{p}\left(z\right)\right)}^{2}}, \end{array}$$

(31) $$\begin{array}{c} \sigma_{q}\left(z\right)=\sqrt{\frac{1}{K}\sum_{i_{s}=1}^{L}{\left(Q\left(i_{s},z\right)-\bar{q}\left(z\right)\right)}^{2}}. \end{array}$$

The Euclidean distance *d* in the PCA space between $\bar{p}\left(z\right)$ and $\bar{q}\left(z\right)$ with selected number of PCs $S^{\prime }$ then can be described as:

(32) $$d=\sqrt{\sum_{z=1}^{S^{\prime }}{\left(\bar{p}\left(z\right)-\bar{q}\left(z\right)\right)}^{2}}.$$

Considering also the obtained standard deviations and subsequent error propagation, the standard deviation $\sigma_{d}$ of *d* can be expressed as:

(33) $$\sigma_{d}=\frac{1}{d}\sqrt{\sum_{z=1}^{S^{\prime }}{\left(\bar{p}\left(z\right)-\bar{q}\left(z\right)\right)}^{2}\left(\sigma_{p}{\left(z\right)}^{2}+\sigma_{q}{\left(z\right)}^{2}\right)}.$$

## 4. Results and Discussion

**Figure 4:**
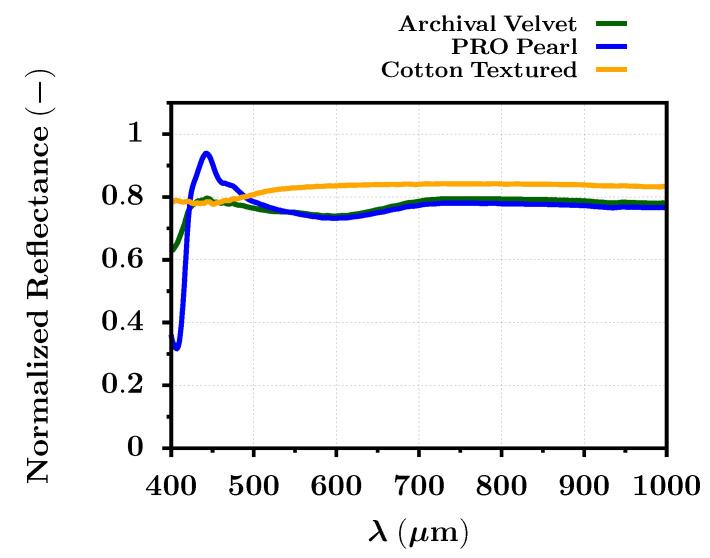
The reflected spectral signature in the VNIR spectral range for the three different blank photo papers extracted from their HS image (FOMEI Archival Velvet, FOMEI PRO Pearl, and FOMEI Cotton Textured).

As was previously mentioned, the reflectance analysis of the dye- and pigment-based ink photographs was performed on three different types of photo paper. However, only the blank photo papers were captured by the VNIR HS camera as a first step. Then, the average reflectance was extracted and analyzed across the whole HS image directly according to Equation (12). The results for all papers are shown in the whole VNIR spectral range in Figure 4. One can observe that the reflected spectra from the PRO Pearl and Archival Velvet paper are considerably similar. This can be due to their semigloss character and the content of optical brighteners in the paper layer. The presence of optical brighteners within the selected papers creates a notable spectral reflectance peak around 440 nm. This is inflicted by the illumination of the paper, which initiates a fluorescence process. The overall photograph then seems whiter and brighter. Due to the fluorescence, the reflectance values may even exceed the level of one near the wavelength of 440 nm. However, the spectral response of the optical brighteners is affected by the spectral distribution of the light source, especially in the ultraviolet (UV) spectral band. The higher amount of UV light incident on the photo paper with optical brighteners, a richer spectral response can be observed.

The influence of the optical brighteners is mainly seen by the reflectance curve of the PRO Pearl paper, which contains significantly more optical brighteners than the Archival Velvet photo paper. On the contrary, the Cotton Textured paper is perfectly matte and does not contain any optical brighteners. Thus, the reflected spectra of the cotton paper moves around the value 0.8 for the whole VNIR spectral range. The presented results also confirmed and quantified the spectral similarity metrics SIDSAM and JMSAM, which are summarized in Table 2 and Table 3.

Regarding the SIDSAM metric, the reflectance comparison of the Archival Velvet photo paper against the PRO Pearl and Cotton Textured photo papers gave scores of 2.9 and 7.9, respectively. The SIDSAM score for the reflectance comparison between the blank Cotton Textured paper and PRO Pearl paper equaled 26.9. On the contrary, the JMSAM metric gave almost similar results for the reflectance comparison between Archival Velvet and the remaining papers PRO Pearl and Cotton Textured with scores of 6.6 × 10${}^{-4}$ and 6.3 × 10${}^{-4}$, respectively. However, the JMSAM metric still evaluated with a higher score (16 × 10${}^{-4}$) the difference between the Cotton Texture and PRO Pearl paper. It was apparent that the most significant difference between the blank paper reflectance was between wavelengths of 400 nm and 550 nm due to the mentioned fluorescence process of the embedded optical brighteners within the layers of the photo paper. With further analysis and more HS image data, it may be even possible to evaluate the amount of brighteners within the paper or photograph and automatically assess the photographic quality.

**Figure 5:**
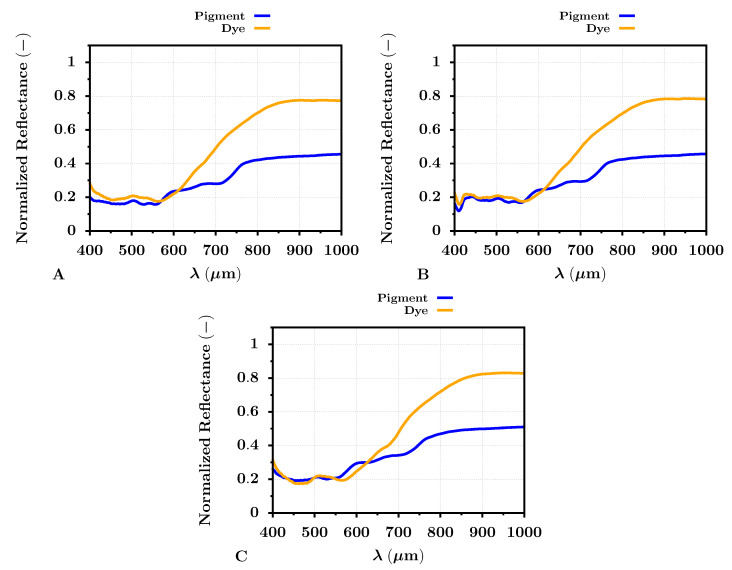
Average VNIR band reflectance of dye- (orange) and pigment-based (blue) test image prints regarding the Archival Velvet (**A**), PRO Pearl (**B**), and Cotton Textured (**C**) photo papers.

The next phase proceeded with fully printed dye- or pigment-based test images on all selected photo papers. These photograph prints were recaptured by the HS camera, with the earlier-mentioned settings. Having all printed testing images, the overall average reflectance (Equation (12)) was extracted from the defined sections of the HS images (see Figure 2). The extracted average reflectance is shown for all photo papers in Figure 5. There was an apparent difference between the dye- and pigment-based ink reflectance values, similar for all selected photo papers. Concerning the dye-based prints, the reflectance increased almost up to a value of 0.8, especially in the spectral band from 800–1000 nm. However, the reflectance of pigment-based inks in the same spectral band did not exceed the level of 0.6. The rise of the dye-based print reflectance started around red colors (around 650 nm). In the band from 400–650 nm, the reflectance of dye- and pigment-based prints was more similar for all papers. The dissimilarity of the average reflectance for dye- and pigment-based prints also supported the spectral metrics. The results of these metrics between the average dye- and pigment-based print reflectance for all photo papers can be seen in Table 4. The SIDSAM metric implied that the difference between the average dye- and pigment-based print reflectance was significant, especially on the PRO Pearl photo paper, with a score of 8.2. For the case of the Archival Velvet photo paper and Cotton Textured photo paper, the scores were 3.2 and 3.1, respectively. According to the SIDSAM metric, the dye- and pigment-based print reflectances were most similar for the prints on the Cotton Textured photo paper, but the spectral difference was still significant. Some influences may also have optical brighteners omitted in the Cotton Textured paper, and therefore, the subsequent fluorescence did not affect the measured reflectance of the inkjet prints. On the contrary, the JMSAM score gave approximately similar results for dye- and pigment-based print reflectance on all selected photo papers. The JMSAM scores were equal to 9.5 × 10${}^{-4}$, 11.0 × 10${}^{-4}$, and 10.0 × 10${}^{-4}$ for the Archival Velvet, PRO Pearl, and Cotton Textured paper, respectively. Therefore, the differences between the dye- and pigment-based prints were also confirmed by the spectral metrics.

Subsequently, the selected color targets or samples (red, green, blue, and black) from the test image (Figure 2) were marked for a similar reflectance assessment and comparison between dye- and pigment-based inks. From each color target area of the HS images, the average reflectance pertaining to the selected colors was extracted and plotted. For simplification, only two distinct photo papers, FOMEI PRO Pearl and FOMEI Cotton Textured, were selected.

**Figure 6:**
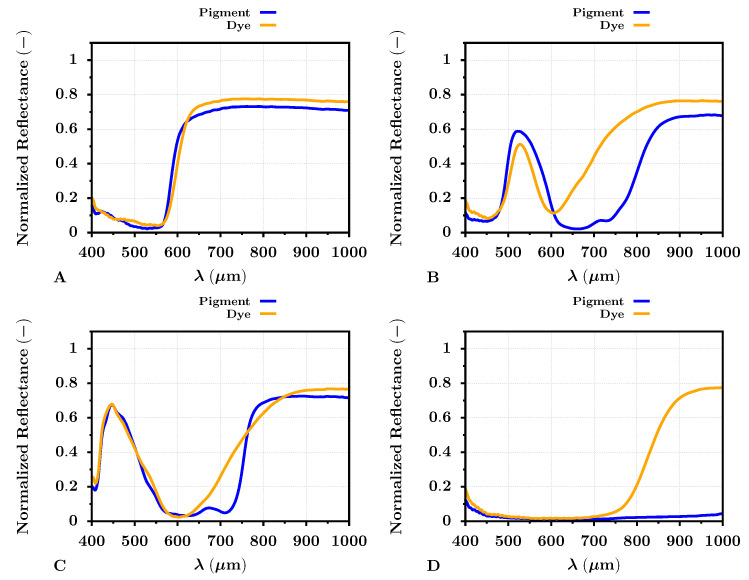
Average color VNIR band reflectance of dye- (orange) and pigment-based (blue) prints on the PRO Pearl inkjet photo paper. The letters (**A**–**D**) represent colors red, green, blue, and black, respectively.

The extracted reflectances of specific colors (red, green, blue, and black) for the PRO Pearl photo paper are shown in Figure 6. The significant differences between dye- and pigment-based printed targets were apparent for all selected colors, but mainly for green and black. The red color target reflected the radiation almost similarly for both types of inks across the VNIR spectrum. The same may be observed for the blue ink color target, where the difference between the dye- and pigment-based targets was apparent in the band from 600–800 nm. The reflectance difference in this spectral band was even more visible for the green color ink targets, where the dye-based green target reflected significantly more radiation than the pigment-based one. The NIR spectral band appeared to be the most important for the black printed color target. The reflectance of the pigment-based black target was low (near the zero level) across the whole VNIR spectrum. Contrary to that, the reflectance of the dye-based black target increased almost up to the level of 0.8 in the NIR spectral band. This increase was apparent approximately from 800 nm. Therefore, the pigment-based black ink almost ideally reflected the radiation in the VNIR spectrum compared to the dye-based ink.

**Figure 7:**
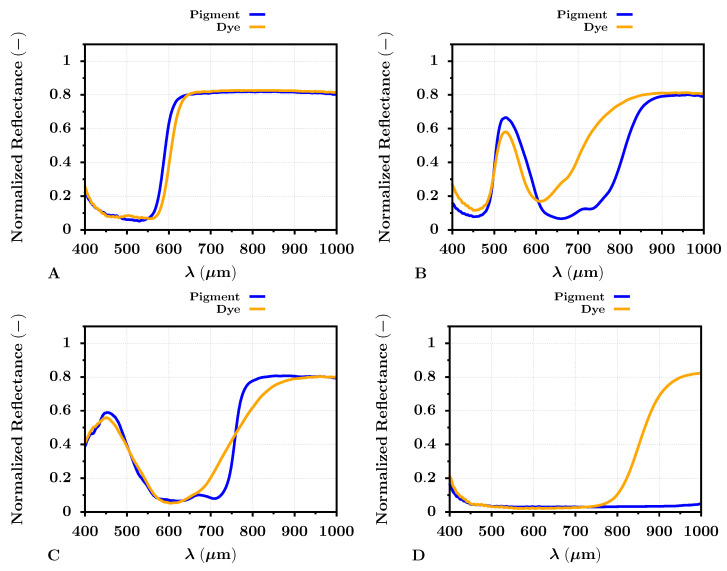
Average color VNIR band reflectance of dye- (orange) and pigment-based (blue) prints on the Cotton Textured inkjet art paper. The letters (**A**–**D**) represent colors red, green, blue, and black, respectively.

Almost similar results were given by the reflectance analysis of the dye- and pigment-based print color targets for the Cotton Textured inkjet art paper. The results can be seen in Figure 7. The reflectance results differed between the selected photo papers, especially for the blue color target, where the blue reflectance peak was at 450 nm, broader than the blue target reflectance peak for the PRO Pearl paper. Again, this can be caused by the optical brighteners in the PRO Pearl photo paper and subsequent fluorescence. However, the difference between the reflectances of the dye- and pigment-based print color targets did not appear to differ from the results for the PRO Pearl paper significantly. The notable spectral difference among the green, black, and blue color targets remained.

The presented graph results of the dye- and pigment-based color targets also supported the selected spectral metrics, the SIDSAM and JMSAM (see Table 5). The VNIR spectral similarity between the red color targets, either dye- or pigment-based, was evaluated by the SIDSAM metric with scores of 0.4 and 1.6 for the PRO Pearl and Cotton Textured photo papers, respectively. The JMSAM gave for the red color target scores of 3.6 × 10${}^{-6}$ and 6.2 × 10${}^{-7}$ for these types of photo papers. These results suggested the mentioned similarity between the dye- and pigment-based red color print targets. The results of the SIDSAM spectral metric for green and blue target prints corresponded to 134 and 10.9 for the PRO Pearl photo paper and 85 and 6.1 for the Cotton Textured photo paper. Similarly, the results of the JMSAM metric for green dye or pigment-based VNIR reflectance comparison were 4.7 × 10${}^{-4}$ and 2.7 × 10${}^{-4}$ for the photo papers, in the same order. The obtained JMSAM score for the dye- and pigment-based print blue target comparison was 2.7 × 10${}^{-5}$ and 1.9 × 10${}^{-5}$ for targets printed on the PRO Pearl and Cotton Textured paper. The spectral metrics also supported the significant difference between the black print targets. The SIDSAM and JMSAM difference score for the targets printed on PRO Pearl photo paper was 547 and 2.2 × 10${}^{-2}$. For the targets printed on the Cotton Textured photo paper, the SIDSAM and JMSAM score was 870 and 2.2 × 10${}^{-2}$, respectively. Especially the score values between the black dye- and pigment-based inks were essential and at least for the JMSAM score, two orders greater than for the remaining color targets. This result indicated a significant dissimilarity between the dye- and pigment-based black ink. This dissimilarity then may serve, for example, a task such as hyperspectral-based automatic ink classification and specific printer identification.

### PCA Analysis

**Figure 8:**
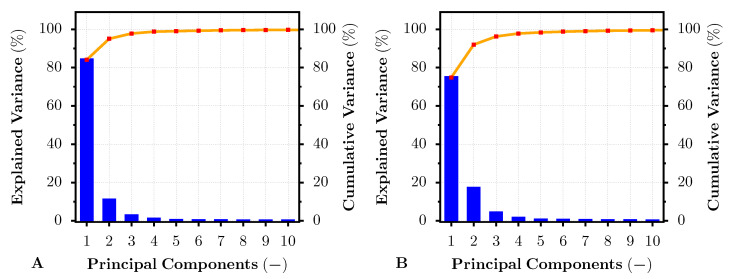
Explained and cumulative variance for the first 10 principal components of PCA-transformed pigment-based (**A**) and dye-based (**B**) inkjet-printed HS image. The result correspond to the dye- and pigment-based test prints on the PRO Pearl inkjet photo paper.

**Figure 9:**
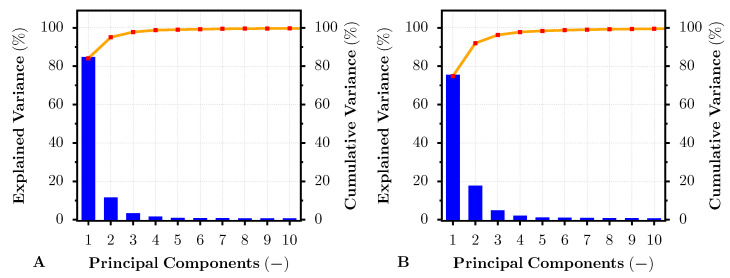
Explained and cumulative variance for the first 10 principal components of PCA-transformed pigment-based (**A**) and dye-based (**B**) inkjet-printed HS image. The result correspond to the dye- and pigment-based test prints on the Cotton Textured inkjet art paper.

In addition, the PCA method was selected for advanced dye- and pigment-based print color target difference analysis. Similarly, as in the previous reflectance analysis, the test image, dye- and pigment-based, was printed on all mentioned photo papers and subsequently captured via the HS camera. The pixel area within the HS image that served for the average dye- and pigment-based reflectance analysis (see Figure 2) was selected as an input for the hyperspectral PCA. The input data were decorrelated and transformed into a set of new orthogonal bases. The HS image then may be expressed by fewer PCs (channels) than the original HS image. Moreover, the variance of the data concerning such PCs can also be easily described. The result of the explained variance from 0–100 % that was covered by the first 10 PCs is introduced in Figure 8 and Figure 9, for the images printed on the PRO Pearl and Cotton Textured paper, respectively. For both of these figures, Subfigure A represents the explained variance pertaining to the specific principal component of the HS image data taken from the pigment-based prints. Complementarily, the subfigures with label B present the explained variance for the PCs obtained from the HS image data of the dye-based prints. From both figures, it is apparent that the first PC generated from the image data of the pigment-based prints covered about 5 % more variance than the first PC of the dye-based print image data. Thus, the variance of the dye-based print images was scattered into more PCs, especially in the second PC. As also shown by the cumulative variance curve within the figures, the images printed by the professional pigment-based printer may be described with fewer PCs, covering 99 % of the total data variance, than the images printed by the hobby dye-based printer. This result seemed to be independent of the photo paper.

During the PCA analysis process, unique eigenvectors were generated from the input image data. These generated eigenvectors may be subsequently used for the new input image data transformation process. This process then transforms the new image input data into a coordinate system based on the previously generated eigenvectors. The transformation may be described according to Equations (26) and (27). Thus, the dye- and pigment-based print images, or more precisely the pixels with spectral values, can be compared within one coordinate system.

**Figure 10:**
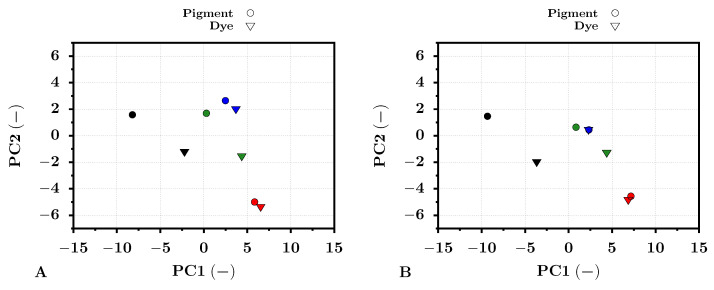
The results of averaged pixel values of the print color targets transformed by the PCA method into the pigment-based coordinate system for dye (triangles) and pigment-based (circles) prints. The results are shown for the first two principal components PC1 and PC2. The point color represents the particular color target. Subfigure (**A**) represents the results for the test images printed on the PRO Pearl photo paper. Subfigure (**B**) presents the results for the test images printed on the Cotton Textured art paper.

Assume a pigment-based test photo print taken by the HS camera. Then, suppose the PCA procedure that exploits the spectral pixels from the created HS image as an input. The eigenvectors generated by this PCA procedure then can be used for the projection of the new input image data of a dye-based photo print into the original pigment-based PCA space. Selecting a specific set of pixels from a specific image location for both transformed images, for example from a particular color target pixel area (see Figure 2), the values of these pixels can be easily compared within this space. For the comparison of the color targets within the pigment-based transformed space, Figure 10 was selected. For this purpose, the same color targets as in the previous analysis, red, green, blue, and black, were selected. The transformed pixel values of these color targets were averaged (see Equation (28)) and plotted. The standard deviations of each averaged point were also computed, but due to the low values and for visual clearness, they were omitted from the presented graphs. The figures were generated separately for the PRO Pearl and Cotton Textured photo papers. For simplicity, only the first two principal components (PC1, PC2) that covered most of the variance were selected. One can see that the color target values for dye- (triangle points) and pigment-based (circle points) inks confirmed the previous reflectance analysis for both types of photo papers. The most significant distances were observable for the pair of black color targets, followed by the green color targets. The red and blue color target pair points, as their reflectances, were positioned considerably closer together, which indicated a high similarity. These results were similar for all photo papers used.

The presented results were also supported by the calculated Euclidean distance *d* (see Equation (32)) between the color target pairs (dye and pigment) across the first 20 PC pigment-based PCA-transformed space. The results can be seen in Table 6. The distances between the red, green, blue, and black targets for the PRO Pearl photo paper were 1.2, 6.25, 2.42, and 9.88, respectively. The distances between the red, green, blue, and black targets for the Cotton Textured paper were equal to 1.45, 5.7, 2.03, and 9.55, respectively. The significant difference between the dye- and pigment-based print color targets was also supported by the standard deviation $\sigma_{d}$ of the obtained distances. For all distances, $d>>3\cdot \sigma_{d}$ applied. The results for the Archival Velvet paper confirmed the presented conclusions. Therefore, the calculated distance confirmed the significant differences between the dye- and pigment-based print color targets in one common coordinate system.

In general, both the reflectance and PCA analysis confirmed that dye- and pigment-based prints might be recognized using the hyperspectral camera in the VNIR spectral band. Thus, identifying original pigment-based and forged dye-based documents, art works, or unprofessionally made photographs printed for archiving purposes is possible. In addition, according to the presented results, having a broader database of ink reflectance pertaining to a particular printer, the automatic classification down to the specific printer seems promising. In the future, a broader spectral band, such as UV or SWIR, should be tested for hyperspectral imaging and ink identification and analysis. Especially, the differences between the dye- and pigment-based inks in the SWIR band may become even more eminent. The future focus should also be on the ink and printer spectral database creation and the design of an automatic ink identification algorithm based on spectral signatures. This classification task then may be exploited for counterfeit and forgery exposure. However, in summary, the most significant result is the confirmation that VNIR HSI can be exploited for dye- and pigment-based ink identification with high fidelity and can validate a similar spectral signature of unknown art works and documents.

## 5. Conclusions

This paper presented the assessment of the spectral characteristics of dye- and pigment-based inkjet prints via VNIR HSI. For the evaluation, the specific test images were printed by the hobby dye-based printer EPSON L1800 and the professional pigment-based printer EPSON SC-P9500. For both selected printers, the test images were printed on three different types of photo paper to cover the variation in the spectral response of the prints. The spectral differences between the dye- and pigment-based prints were assessed via the plotted reflectance and quantified via the selected spectral similarity metrics SIDSAM and JMSAM. The spectral difference measurement was performed separately for the whole printed image and the specific printed color targets. The spectral differences between the printed color targets were also assessed via PCA. It was shown that the overall average reflectance of the pigment-based print in the NIR spectral band was significantly lower than the dye-based one, regardless of the exploited photo paper. The most significant spectral difference was observed for the black printed color target. This was also confirmed by the subsequent PCA procedure applied to the acquired HS image data. With a broader HS database of either dye- or pigment-based prints, an advanced classification algorithm based on the captured spectral data may be developed. Therefore, the presented analysis could be an important step toward the automatic hyperspectral-based classification of inks, whole prints, and even specific printers, and it may lead to a reliable print forgery identification.

## Figures and Tables

**Table 1:** Parameters of the SPECIM PFD4K-65-V10E hyperspectral camera.

Spectral range	400–1000 nm
Spectral resolution FWHM	3.0 nm
Spectral sampling	0.78–6.27 nm/pixel
Spatial resolution RMS spot size	<9 μm
Focal length	23 mm
F-number	F/2.4
Slit width	30 μm
Effective slit length	14.2 mm
Total efficiency (typical)	>50 % (polar. ind.)
Detector	CMOS
Spatial pixels	1775
Spectral bands	768
Pixel size	8.0 × 8.0 μm

**Table 2:** The SIDSAM score of the spectral comparison between the VNIR reflectance of blank photo papers FOMEI Archival Velvet, FOMEI PRO Pearl, and FOMEI Cotton Textured.

	Archival Velvet	PRO Pearl	Cotton Textured
Archival Velvet	0.0	2.9	7.9
PRO Pearl	2.9	0.0	26.9
Cotton Textured	7.9	26.9	0.0

**Table 3:** The JMSAM score of the spectral comparison between the VNIR reflectance of blank photo papers FOMEI Archival Velvet, FOMEI PRO Pearl, and FOMEI Cotton Textured.

	Archival Velvet	PRO Pearl	Cotton Textured
Archival Velvet	0.0	6.6 × 10${}^{-4}$	6.3 × 10${}^{-4}$
PRO Pearl	6.6 × 10${}^{-4}$	0.0	16 × 10${}^{-4}$
Cotton Textured	6.3 × 10${}^{-4}$	16 × 10${}^{-4}$	0.0

**Table 4:** The spectral metrics values of the extracted average reflectance of dye- vs. pigment-based prints.

	SIDSAM	JMSAM
Archival Velvet	3.1	9.5 × 10${}^{-4}$
PRO Pearl	8.2	11.0 × 10${}^{-4}$
Cotton Textured	3.2	10.0 × 10${}^{-4}$

**Table 5:** Dye- vs. pigment-based reflectance comparison for the specific color selection.

	Red		Green		Blue		Black	
	SIDSAM	JMSAM	SIDSAM	JMSAM	SIDSAM	JMSAM	SIDSAM	JMSAM
Archival Velvet	0.4	2.9 × 10${}^{-6}$	119	5.0 × 10${}^{-4}$	11.1	2.3 × 10${}^{-5}$	612	2.1 × 10${}^{-2}$
PRO Pearl	0.5	3.6 × 10${}^{-6}$	134	4.7 × 10${}^{-4}$	10.9	2.7 × 10${}^{-5}$	547	2.2 × 10${}^{-2}$
Cotton Textured	1.6	6.2 × 10${}^{-7}$	85	2.9 × 10${}^{-4}$	6.1	1.9 × 10${}^{-5}$	870	2.2 × 10${}^{-2}$

**Table 6:** Euclidean distance *d* and its standard deviation $\sigma_{d}$ between the averaged pixel values of the dye- and pigment-based print color targets. The pixel values of both type print targets were transformed into the pigment-based PCA coordinate system. The distances were calculated across the first 20 PCs.

	Red		Green		Blue		Black	
	*d*	$\sigma_{d}$	*d*	$\sigma_{d}$	*d*	$\sigma_{d}$	*d*	$\sigma_{d}$
Archival Velvet	1.13	0.11	6.19	0.08	2.42	0.08	10.71	0.08
PRO Pearl	1.2	0.12	6.25	0.08	2.42	0.12	9.88	0.12
Cotton Textured	1.45	0.18	5.7	0.19	2.03	0.1397	9.55	0.11

## Data Availability

The data presented in this study are available from the corresponding author on request.
